# Enhanced muscarinic M1 receptor gene expression in the corpus striatum of streptozotocin-induced diabetic rats

**DOI:** 10.1186/1423-0127-16-38

**Published:** 2009-04-03

**Authors:** G Gireesh, T Peeyush Kumar, Jobin Mathew, CS Paulose

**Affiliations:** 1 Molecular Neurobiology and Cell Biology Unit, Centre for Neuroscience, Cochin University of Science and Technology, Cochin- 682 022, Kerala, India

## Abstract

Acetylcholine (ACh), the first neurotransmitter to be identified, regulate the activities of central and peripheral functions through interactions with muscarinic receptors. Changes in muscarinic acetylcholine receptor (mAChR) have been implicated in the pathophysiology of many major diseases of the central nervous system (CNS). Previous reports from our laboratory on streptozotocin (STZ) induced diabetic rats showed down regulation of muscarinic M1 receptors in the brainstem, hypothalamus, cerebral cortex and pancreatic islets. In this study, we have investigated the changes of acetylcholine esterase (AChE) enzyme activity, total muscarinic and muscarinic M1 receptor binding and gene expression in the corpus striatum of STZ – diabetic rats and the insulin treated diabetic rats. The striatum, a neuronal nucleus intimately involved in motor behaviour, is one of the brain regions with the highest acetylcholine content. ACh has complex and clinically important actions in the striatum that are mediated predominantly by muscarinic receptors. We observed that insulin treatment brought back the decreased maximal velocity (V_max_) of acetylcholine esterase in the corpus striatum during diabetes to near control state. In diabetic rats there was a decrease in maximal number (B_max_) and affinity (K_d_) of total muscarinic receptors whereas muscarinic M1 receptors were increased with decrease in affinity in diabetic rats. We observed that, in all cases, the binding parameters were reversed to near control by the treatment of diabetic rats with insulin. Real-time PCR experiment confirmed the increase in muscarinic M1 receptor gene expression and a similar reversal with insulin treatment. These results suggest the diabetes-induced changes of the cholinergic activity in the corpus striatum and the regulatory role of insulin on binding parameters and gene expression of total and muscarinic M1 receptors.

## Background

The most and well known effects of diabetes mellitus on CNS is dysfunction of neurotransmitters, which is secondary to the metabolic disorders such as hyperglycemia and acidosis. It has been proposed that an unbalanced autonomic nervous system may be a major cause of the metabolic syndrome [1] Diabetes mellitus have also been reported to be accompanied by a number of behavioral and hormonal abnormalities, including hyperphagia, reduced motor activity [2,3]. CNS abnormalities including neuronal atrophy and axonal degenerations [4,5] are also associated with diabetes. The altered levels of neurotransmitter in specific brain areas in patients with diabetes mellitus [6] and in animals with experimental diabetes [7-12] have been documented and implicated in the CNS disorders. ACh, a major neurotransmitter from autonomic nervous system, regulates the cholinergic stimulation of insulin secretion, through interactions with muscarinic receptors. Recently we have reported that muscarinic M1 receptor gene expressions were decreased in the cerebral cortex, brainstem, hypothalamus and pancreatic islets of STZ induced diabetic rats and insulin modulates the binding parameters and gene expression [13,14]. Acetylcholine muscarinic receptors, members of the superfamily of G protein-coupled receptors, are classified pharmacologically into M_1 _to M_5 _subtypes, with M_1_, M_3 _and M_5 _receptors preferentially coupling to Gα_q/11 _proteins and M_2 _and M_4 _receptors to Gα_i/o _proteins [9]. All five muscarinic receptors are expressed by striatal neurones, with M_1 _and M_4 _receptors as the predominant subtypes, conforming together nearly 80% of the receptor population in the rat as shown by immunodetection [15,16] The main objective of the present study was to determine whether uncontrolled hyperglycemia, as a consequence of diabetes, altered the acetylcholine esterase enzyme activity, total and muscarinic M1 receptor binding parameters and muscarinic M1 receptor gene expression and the regulatory role of insulin in the rat corpus striatum.

## Materials and methods

Biochemicals used in the present study were purchased from Sigma Chemical Co., St. Louis, USA. All other reagents were of analytical grade purchased locally. Quinuclidinyl benzilate, L- [Benzilic-4,4'-^3^H], ([^3^H] QNB)(Sp. Activity 42 Ci/mmol) was purchased from NEN life sciences products Inc., Boston, U.S.A. Tri-reagent kit was purchased from MRC, USA. Real Time PCR Taqman probe assays on demand were purchased from Applied Biosystems, Foster City, CA, USA.

Male adult Wistar rats of 180–240 g body weight were used for all experiments. They were housed in separate cages under 12 hour light and 12 hour dark periods. Rats have free access to standard food and water ad libitum. All animal care and procedures were in accordance with the Institutional and National Institute of Health guidelines. Diabetes was induced in rats by single intrafemoral injection of streptozotocin freshly dissolved in 0.1 M citrate buffer, pH 4.5, under anesthesia [17]. Streptozotocin was given at a dose of 55 mg/Kg body weight [18,19]. Animals were divided into the following groups: i) Control ii) diabetic iii) insulin-treated diabetic rats. Each group consisted of 6–8 animals. The insulin-treated diabetic group received subcutaneous injections (1 Unit/kg body weight) of Lente and Plain insulin (Boots India) daily during the entire period of the experiment. The last injection was given 24 hrs before sacrificing the rats. Rats were sacrificed on 15^th ^day by decapitation. The corpus striatum was dissected out quickly over ice according to the procedure of Glowinski and Iversen 1966[20], the tissues were stored at -70°C until assayed.

### Estimation of blood glucose

Blood glucose was estimated by the spectrophotometric method using glucose oxidase-peroxidase reactions. Blood samples were collected from the tail vein at 0 hours (Before the start of the experiment), 3^rd ^day, 6^th ^day, 10^th ^day and 14^th ^day and the glucose levels were estimated. Blood samples were collected 3 hrs after the administration of morning dose. The results were expressed in terms of milligram per deciliter of blood.

### Acetylcholine Esterase Assay

Acetylcholine esterase asssay was done using the spectrophotometric method of Ellman et al, (1961) [21]. The corpus striatum homogenate (10%) was prepared in sodium phosphate buffer (30 mM, pH-7). One ml of 1% Triton × 100 was added to the homogenate to release the membrane bound enzyme and centrifuged at 10,000 × g for 30 minutes at 4°C. Different concentrations of acetylthiocholine iodide were used as substrate. The mercaptan formed as a result of the hydrolysis of the ester reacts with an oxidising agent 5,5'-dithiobis (2-Nitrobenzoate) absorbs at 412 nm.

### Total Muscarinic and muscarinic M1 receptor binding studies in the corpus striatum

[^3^H]QNB binding assay in corpus striatum was done according to the modified procedure of Yamamura and Snyder (1981) [22]. Corpus striatum was homogenised in a polytron homogeniser with 20 volumes of cold 50 mM Tris-HCl buffer, containing 1 mM EDTA, pH.7.4. The supernatant was then centrifuged at 30,000 × g for 30 minutes and the pellets were resuspended in appropriate volume of Tris-HCl-EDTA buffer.

Total muscarinic receptor binding parameter assays were done using different concentrations i.e., 0.1–2.5 nM of [^3^H] QNB in the incubation buffer, pH 7.4 in a total incubation volume of 250 μl containing appropriate protein concentrations (200–250 μg). Non-specific binding was determined using 100 μM atropine. Competition studies were carried out with 1 nM [^3^H]QNB in each tube with atropine concentrations varying from 10^-9 ^– 10^-4 ^M atropine. Tubes were incubated at 22°C for 60 minutes and filtered rapidly through GF/C filters (Whatman). The filters were washed quickly by three successive washing with 5.0 ml of ice cold 50 mM Tris-HCl buffer, pH 7.4. Bound radioactivity was counted with cocktail-T in a Wallac 1409 liquid scintillation counter. The non-specific binding determined showed 10% in all our experiments.

Muscarinic M1 receptor binding assays were done using different concentrations i.e., 0.1–2.5 nM of [^3^H]QNB in the incubation buffer, pH 7.4 in a total incubation volume of 250 μl containing appropriate protein concentrations (200–250 μg). Non-specific binding was determined using 100 μM pirenzepine. Competition studies were carried out with 1 nM [^3^H]QNB in each tube with pirenzepine concentrations varying from 10^-9 ^– 10^-4 ^M. Tubes were incubated at 22°C for 60 minutes and filtered rapidly through GF/C filters (Whatman). The filters were washed quickly by three successive washing with 5.0 ml of ice cold 50 mM Tris-HCl buffer, pH 7.4. Bound radioactivity was counted with cocktail-T in a Wallac 1409 liquid scintillation counter. The non-specific binding determined showed 10% in all our experiments.

### Protein determination

Protein was measured by the method of Lowry et al, (1951) [23] using bovine serum albumin as standard. The intensity of the purple blue colour formed was proportional to the amount of protein, which was read in a spectrophotometer at 660 nm

### Receptor data analysis

The receptor binding parameters were determined using Scatchard analysis [24]. The specific binding was determined by subtracting non-specific binding from the total. The binding parameters, maximal binding (B_max_) and equilibrium dissociation constant (K_d_), were derived by linear regression analysis by plotting the specific binding of the radioligand on X-axis and bound/free on Y-axis using Sigma plot software (version 2.0, Jandel GmbH, Erkrath, Germany). The maximal binding is a measure of the total number of receptors present in the tissue and the equilibrium dissociation constant is the measure of the affinity of the receptors for the radioligand. The K_d _is inversely related to receptor affinity.

### Displacement curve analysis

The displacement data were analysed by nonlinear regression using GraphPad PRISM™ software, GraphPad Inc., San Diego, USA. The concentration of the competing drug that competes for half the specific binding was defined as EC_50 _which is same as IC_50_. The affinity of the receptor for the competing drug is designated as K_i _and is defined as the concentration of the competing ligand that will bind to half the binding sites at equilibrium in the absence of radioligand or other competitors. The Hill slope was used to indicate a one or two-sited model of curve-fitting.

### Analysis of gene expression by Real-Time PCR

RNA was isolated from the corpus striatum of experimental rats using the Tri reagent (MRC, USA). Total cDNA synthesis was performed using ABI PRISM cDNA arhive kit in 0.2 ml microfuge tubes. The reaction mixture of 20 μl contained 0.2 μg total RNA, 10 × RT buffer, 25 × dNTP mixture, 10 × random primers, MultiScribe RT (50 U/μl) and RNase free water. The cDNA synthesis reactions were carried out at 25°C for 10 minutes and 37°C for 2 hours using an Eppendorf Personal Cycler. Real-time PCR assays were performed in 96-well plates in ABI 7300 real-time PCR instrument (Applied Biosystems). The primers and probes were purchased from Applied Biosystems, Foster City, California, USA. The TaqMan reaction mixture of 20 μl contained 25 ng of total RNA-derived cDNAs, 200 nM each of the forward primer, reverse primer, and TaqMan probe for Muscarinic M1 receptor gene and endogenous control (β-actin) and 12.5 μl of Taqman 2× Universal PCR Master Mix (Applied Biosystems) and the volume was made up with RNAse free water. The following thermal cycling profile was used (40 cycles): 50°C for 2 min, 95°C for 10 min, 95°C for 15 sec and 60°C for 1 min.

Fluorescence signals measured during amplification were considered positive if the fluorescence intensity was 20-fold greater than the standard deviation of the baseline fluorescence. The ΔΔCT method of relative quantification was used to determine the fold change in expression. This was done by first normalizing the resulting threshold cycle (CT) values of the target mRNAs to the CT values of the internal control β-actin in the same samples (ΔCT = CT_Target _– CT_β-actin_). It was further normalize with the control (ΔΔCT = ΔCT – CT_Control_). The fold change in expression was then obtained as (2^-ΔΔ^CT) and the graph was plotted using log 2^-ΔΔ^CT.

### Statistics

Statistical evaluations were done by ANOVA, expressed as mean ± S.E.M using InStat (Ver.2.04a) computer programme.

## Results

Blood glucose level of all rats before streptozotocin administration was within the normal range. Streptozotocin administration led to a significant increase (p < 0.001) in blood glucose level of diabetic rats when compared to control rats. Insulin treatment was able to significantly reduce (p < 0.001) the increased blood glucose level to near the control value when compared to diabetic group (Table 1)

**Table 1:** Blood glucose (mg/dl) level in Experimental rats

Animal status	0 day (Before STZ injection)	3^rd ^day (Initial)	6^th ^day	10^th ^day	14^th ^day (Final)
Control	86.2 ± 1.4	93.5 ± 1.6	89.4 ± 0.8	101.2 ± 2.2	97.7 ± 1.21
Diabetic	79.4 ± 1.5	253.1 ± 0.5	303.1 ± 0.8	309.7 ± 0.6	311.9 ± 1.4***
D + I	85.2 ± 0.8	256.8 ± 0.5	303.6 ± 0.7	190.9 ± 1.5	137.0 ± 1.3^ψψψφφφ^

Values are mean ± S.E.M of 4–6 rats in each group. Each group consist of 6–8 rats

*** P < 0.001 when compared to control, ^ψψψ ^P < 0.001 when compared to diabetic group, φφφ p < 0.001 when compared with initial reading

### Acetylcholine esterase activity in the Corpus striatum of experimental rats

Acetylcholine esterase kinetics studies showed that V_max _was significantly decreased (p < 0.001) in the corpus striatum of diabetic group with no significant change in K_m_. Insulin treatment significantly reversed the V_max _(p < 0.001) to near control value when compared to diabetic group (Table 2).

**Table 2:** Acetylcholine esterase activity in the corpus striatum of Control, Diabetic and D+I group rats

Animal status	Vmax (μmoles/min/mg protein)	Km (μM)
Control	2420.0 ± 11.5	51.0 ± 0.5

Diabetic	1631.3 ± 16.1***	51.3 ± 0.8

Diabetic + Insulin treated (D+I)	2647.0 ± 26.5^ψψψ^	51.0 ± 0.5

Values are mean ± S.E.M of 4–6 separate experiments. Each group consist of 6–8 rats

*** P < 0.001 when compared to control, ^ψψψ ^P < 0.001 when compared to diabetic group

### Total Muscarinic receptor analysis

#### Scatchard analysis of [^3^H] QNB binding against atropine in the corpus striatum of Control, Diabetic and Diabetic+Insulin treated diabetic rats

**Figure 1:**
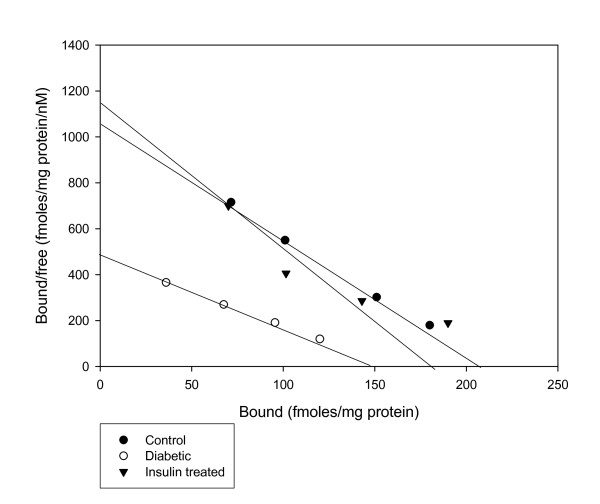
**Representative graph showing Scatchard analysis of [^3^H]QNB binding against atropine in the corpus striatum of Control, Diabetic and Diabetic+Insulin treated group rats**. Control (black circle), Diabetic (open circle), Insulin treated diabetic rats (black triangle). Total muscarinic receptor binding parameter assays were done using different concentrations i.e., 0.1–2.5 nM of [^3^H]QNB in the incubation buffer, pH 7.4 in a total incubation volume of 250 μl containing appropriate protein concentrations (200–250 μg). Non-specific binding was determined using 100 μM atropine. Tubes were incubated at 22°C for 60 minutes and filtered rapidly through GF/C filters (Whatman). The filters were washed quickly by three successive washing with 5.0 ml of ice cold 50 mM Tris-HCl buffer, pH 7.4. Bound radioactivity was counted with cocktail-T in a Wallac 1409 liquid scintillation counter. The non-specific binding determined showed 10% in all our experiments.

The Scatchard analysis showed that the B_max _and K_d _of the [^3^H]QNB receptor binding decreased significantly (p < 0.001) in the corpus striatum of diabetic rats when compared to control group. In insulin treated diabetic group B_max _and K_d _were significantly (p < 0.001) reversed back to near control value when compared to diabetic group. (Fig 1 & Table 3)

**Table 3:** Scatchard analysis of [^3^H] QNB binding against atropine in the corpus striatum of Control, Diabetic, and Diabetic+Insulin treated group rats

Animal status	B_max_ (fmoles/mg protein)	K_d _(nM)
Control	214.00 ± 3.05	0.19 ± 0.01

Diabetic	150.00 ± 5.77***	0.27 ± 0.05***

Diabetic + Insulin treated	184.00 ± 3.05^ψψψ^	0.17 ± 0.05^ψψψ^

Values are mean ± S.E.M of 4–6 separate experiments. Each group consist of 6–8 rats

*** P < 0.001 when compared to control, ^ψψψ ^P < 0.001 when compared to diabetic group

#### Displacement analysis of [^3^H]QNB using Atropine

**Figure 2:**
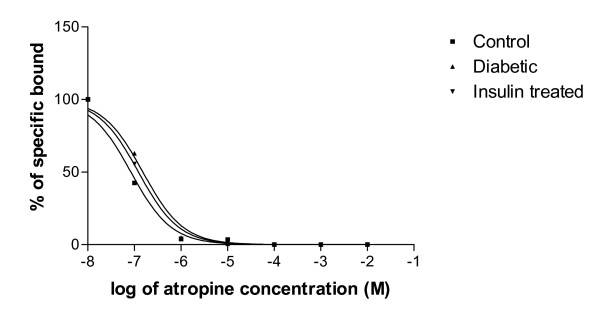
**Representative graph showing displacement analysis of [^3^H]QNB binding against atropine in the hypothalamus of Control, Diabetic and Diabetic+Insulin treated group rats**. Control (black circle), Diabetic (upward pointing black triangle), Insulin treated diabetic rats (downward pointing black triangle). Competition studies were carried out with 1 nM [^3^H]QNB in each tube with atropine concentrations varying from 10^-9^, 10^-4 ^M. Data were fitted with iterative nonlinear regression software (Prism, GraphPad, San Diego, CA). K_i _The affinity of the receptor for the competing drug. EC_50 _is the concentration of the competitor that competes for half the specific binding.

In the displacement analysis, the competitive curve fitted to a one-sited model in all groups with Hill slope values were near to unity. The log (EC_50_) did not alter in all the experimental groups. The Ki decreased in diabetic condition (Fig 2 & Table 4).

**Table 4:** Binding parameters of [^3^H] QNB against atropine in the corpus striatum of Control, Diabetic and Diabetic+Insulin treated group rats

Experimental Group	Best-fit model	Log (EC_50_)	Ki	Hill slope
Control	One-site	-7.076	2.10 × 10^-8^	0.9832

Diabetic	One-site	-6.818	3.80 × 10^-8^	0.9883

Insulin treated diabetic	One-site	-6.913	3.05 × 10^-8^	0.9889

Values are mean of 4–6 separate experiments. Each group consist of 6–8 rats

### Muscarinic M1 receptor analysis

#### Scatchard analysis of [^3^H]QNB binding against pirenzepine in the corpus striatum of Control, Diabetic, and Diabetic+Insulin treated diabetic rats

**Figure 3:**
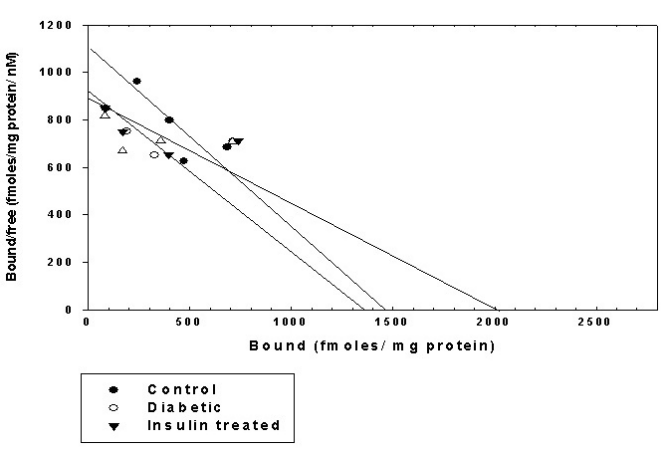
**Representative graph showing Scatchard analysis of [^3^H]QNB binding against pirenzepine in the corpus striatum of Control, Diabetic and Diabetic+Insulin treated group rats**. Control (black circle), Diabetic (open circle), Insulin treated diabetic rats (black triangle). Muscarinic M1 receptor binding parameter assays were done using different concentrations i.e., 0.1–2.5 nM of [^3^H]QNB in the incubation buffer, pH 7.4 in a total incubation volume of 250 μl containing appropriate protein concentrations (200–250 μg). Non-specific binding was determined using 100 μM pirenzepine. Tubes were incubated at 22°C for 60 minutes and filtered rapidly through GF/C filters (Whatman). The filters were washed quickly by three successive washing with 5.0 ml of ice cold 50 mM Tris-HCl buffer, pH 7.4. Bound radioactivity was counted with cocktail-T in a Wallac 1409 liquid scintillation counter. The non-specific binding determined showed 10% in all our experiments.

The Scatchard analysis showed that the B_max _of muscarinic M1 receptors of corpus striatum was increased significantly (p < 0.001) in diabetic condition when compared to control group while the K_d _was decreased significantly when compared to control group (p < 0.001). In insulin treated diabetic rats B_max _was significantly (p < 0.001) reversed back to near control value when compared to diabetic group but K_d _was not reversed back to near control value when compared to diabetic group (Fig 3 & Table 5).

**Table 5:** Scatchard analysis of [^3^H] QNB binding against pirenzepine in the corpus striatum of Control, Diabetic and Diabetic+Insulin treated group rats

Animal status	B_max _(fmoles/mg protein)	K_d _(nM)
Control	1460.00 ± 30.55	1.34 ± 0.02

Diabetic	2060.00 ± 30.55***	0.45 ± 0.02***

Diabetic + Insulin treated	1550.00 ± 28.86^ψψψ^	0.56 ± 0.01

Values are mean ± S.E.M of 4–6 separate experiments. Each group consist of 6–8 rats

*** P < 0.001 when compared to control, ^ψψψ ^P < 0.001 when compared to diabetic group

#### Displacement analysis of [^3^H]QNB using pirenzepine

**Figure 4:**
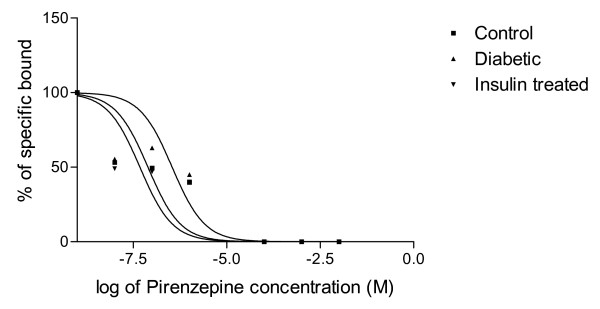
**Representative graph showing displacement analysis of [^3^H]QNB binding against pirenzepine in the corpus striatum of Control, Diabetic and Diabetic+Insulin treated group rats**. Control (black circle), Diabetic (upward pointing black triangle), Insulin treated diabetic rats (downward pointing black triangle). Competition studies were carried out with 1 nM [^3^H]QNB in each tube with pirenzepine concentrations varying from 10^-9 ^to 10^-4 ^M. Data were fitted with iterative nonlinear regression software (Prism, GraphPad, San Diego, CA). K_i _– The affinity of the receptor for the competing drug. EC_50 _is the concentration of the competitor that competes for half the specific binding.

In the displacement analysis, the competitive curve fitted to a one-site model in all the experimental conditions. Hill slopes were near unity confirming the one-site model. There were no changes in the log (EC50) values. The Ki value was decreased in diabetic condition (Fig 4 & Table 6).

**Table 6:** Binding parameters of [^3^H]QNB against pirenzepine in the corpus striatum of Control, Diabetic and Diabetic+Insulin treated group rats

Experimental Group	Best-fit model	Log (EC_50_)	Ki	Hill slope
Control	One-site	-7.107	2.23 × 10^-8^	0.7224

Diabetic	One-site	-6.459	9.92 × 10^-8^	0.7444

Insulin treated diabetic	One-site	-7.322	4.76 × 10^-8^	0.6826

Values are mean of 4–6 separate experiments. Each group consist of 6–8 rats

### Real Time-PCR analysis

**Figure 5:**
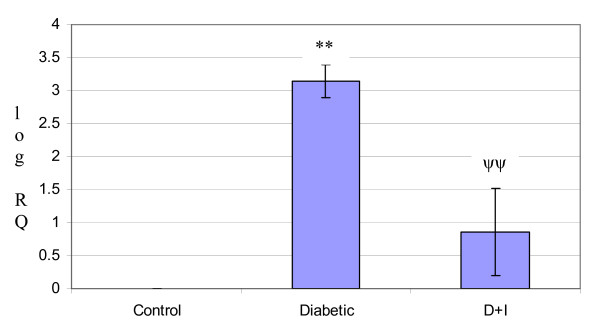
**Representative graph showing Real-Time amplification of muscarinic M1 mRNA from the corpus striatum of control, diabetic and insulin treated diabetic rats**. Control, Diabetic, Insulin treated diabetic rats. The ΔΔCT method of relative quantification was used to determine the fold change in expression. This was done by first normalizing the resulting threshold cycle (CT) values of the target mRNAs to the CT values of the internal control β-actin in the same samples (ΔCT = CT_Target _– CT β-actin). It was further normalize with the control (ΔΔCT = ΔCT – CT_Control_). The fold change in expression was then obtained (2^-ΔΔCT^). The graph was plotted using log 2^-ΔΔCT^. Values are mean ± S.D of 4–6 separate experiments. Relative Quantification values and standard deviations are shown in the table. The relative ratios of mRNA levels were calculated using the ΔΔCT method normalized with β-actin CT value as the internal control and Control CT value as the calibrator.

Real Time-PCR analysis showed that the muscarinic M1 receptor gene expression was increased significantly (p < 0.01) in diabetic condition and it reversed to near control value in insulin treated diabetic rats (Fig 5 & Table 7).

**Table 7:** Real Time amplification of Muscarinic M1 receptor mRNA from the Corpus striatum of Control, Diabetic, and Diabetic+Insulin treated group rats

Experimental group	RQ Value
Control	0

Diabetic	3.14 ± 0.25**

Diabetic + Insulin treated	0.86 ± -0.66^ψψ^

Values are mean ± S.D of 4–6 separate experiments. Each group consist of 6–8 rats Relative Quantification values and standard deviations are shown in the table. The relative ratios of mRNA levels were calculated using the ΔΔCT method normalized with β-actin CT value as the internal control and Control CT value as the calibrator.

** p < 0.01 when compared with control

^ψψ^p < 0.01 when compared with diabetic group

## Discussion

The brain neurotransmitters receptor activity and hormonal pathways control many physiological functions in the body. The present study analyzed the changes of acetylcholine esterase enzyme activity, total muscarinic and muscarinic M1 receptors in the corpus striatum of STZ diabetic and insulin-treated diabetic rats. CNS mAChRs regulate a large number of important central functions including cognitive, behavioural, sensory, motor and autonomic processes [25-27]. A key feature of type 2 diabetes is that pancreatic β-cells fail to release sufficient amounts of insulin despite elevated blood glucose levels [28]. Glucose-stimulated insulin secretion (GSIS) is regulated by numerous hormones and neurotransmitters most of which act on specific G-protein-coupled receptors (GPCRs) expressed by pancreatic β-cells [29,30]. Many studies have shown that ACh, following its release from intra-pancreatic parasympathetic nerve endings, can stimulate β-cell mAChRs, leading to enhanced GSIS in a strictly glucose-dependent fashion [29,30]. mAChRs are members of the superfamily of GPCRs. Molecular-cloning studies have revealed the existence of five molecularly distinct mammalian mAChR subtypes, M1-M5 [31,9]. Earlier studies from our laboratory have established the central neurotransmitter receptor subtypes functional regulation during diabetes, pancreatic regeneration and cell proliferation [32-37]. M_1 _mAChRs are abundantly expressed in all major regions of the forebrain, including striatum, hippocampus, and cerebral cortex [38-40,15]. It is therefore likely that M_1 _mAChRs play a role in the many central actions of ACh that involve the activity of forebrain mAChRs. Pharmacological evidence suggests that M_1 _receptors are involved in mediating higher cognitive processes, such as learning and memory [41,42,28]. The striatum, a neuronal nucleus intimately involved in motor behaviour, is one of the brain regions with the highest acetylcholine content [43]. The mRNA for M1 is present in more than 80% of striatal neurons [44] including cholinergic neurons, substance P neurons, enkephalin neurons, and somatostatin neurons [45]. Recent studies from our laboratory have showed the significance of muscarinic and muscarinic M1 receptors in the cerebral cortex, hypothalamus, brainstem, and pancreatic islets of STZ induced diabetic rats and its functional regulation in insulin secretion. [13,14]. ACh, through vagal muscarinic and non-vagal muscarnic pathways [46] increases insulin secretion [47]. They function through muscarinic receptors present on pancreatic islet cells. Receptor localization studies suggest that multiple muscarinic receptors (M_1_, M_3_, M_4_, and M_5_) are expressed in pancreatic islets/β-cells [48]. From our previous studies it was observed that muscarinic M1 receptors were down-regulated during STZ diabetes [13]. The enzyme AChE indirectly plays an important role in transmission of nerve impulse. It hydrolyses the ACh released at the cholinergic synapse and thus terminates the action of this neurotransmitter. In addition to their role in cholinergic transmission, cholinesterases may also play a role during morphogenesis and neurodegenerative diseases [49,50]. We observed a significant decrease in V_max _of acetylcholine esterase in the striatum of diabetic rats which was reversed to near control level by insulin treatment. Akmayev et al (1978) [51] showed that there is a difference in distribution of enzyme in the neurons of the central vagal nuclei in normal and adult male rats. It is suggested that the changes in the plasma glucose or insulin may be that stimulus that influence the activity of cholinergic neurons. Insulin treatment reversed the altered maximum velocity toward the control level. Corpus striatum is best known for its role in the planning and modulation movement pathways but also involved in a variety of cognitive process involving executive function. In corpus striatum total muscarinic receptor numbers and affinity were decreased during diabetes, whereas muscarinic M1 receptors number was increased in STZ diabetic rats with decrease in affinity. The changes in the receptor number and affinity observed are due to the alterations of receptor protein and synthesis. Real-time PCR analysis showed an up-regulation of the muscarinic M1 receptor mRNA level in the striatum of diabetic rats, whereas it reversed to near control when treated with insulin. This is in accordance with our receptor binding studies. ACh has complex and clinically important actions in the striaturn that are mediated predominantly by muscarinic receptors. Based on physiological and pharmacological studies, several specific actions of ACh in the striatum have been suggested. ACh regulates its own release from cholinergic interneurons through presynaptic autoreceptors. Noncholinergic striatal neurons are directly affected by ACh through postsynaptic receptors and presynaptic heteroceptors and the release of excitatory amino acids and dopamine by extrinsic striatal afferents may be under presynaptic control of ACh through presynaptic heteroceptors [52-54]. In diabetic corpus striatum total muscarinic receptors activities were decreased. The insulin treatment reversed these altered parameters to near control level. Muscarinic receptor subtypes other than M1 may also be affected by the diabetic condition. Further studies have to be carried out to elucidate the role of other subtypes. The present study suggests that drugs that can selectively activate muscarinic receptors may be of significant therapeutic benefit in the diabetes management. Thus our results revealed the significance of central muscarinic receptor changes during diabetes and the regulatory role of insulin on muscarinic receptors.

## Competing interests

The authors declare that they have no competing interests.

## Authors' contributions

GG and CSP designed research; GG performed experiments; PKT and JM helped GG in experiments; GG and CSP analyzed the data; GG and CSP wrote the paper. All authors read and approved the final manuscript.
